# NSF-Based Analysis of the Structural Stressing State of Trussed Steel and a Concrete Box Girder

**DOI:** 10.3390/ma15113785

**Published:** 2022-05-26

**Authors:** Jian Yuan, Jie Lai, Feng Xu, Zhengfa Wu, Suhui Yu, Guorui Sun

**Affiliations:** 1 School of Civil Engineering and Architecture, Wuhan Institute of Technology, Wuhan 430205, China; yuanjian_850809@126.com (J.Y.); dadalai12342022@163.com (J.L.); 13026375729@163.com (Z.W.); 2 Academy of Combat Support, Rocket Force University of Engineering, Xi’an 710025, China; yusuhui88@126.com; 3 School of Transportation Science and Engineering, Harbin Institute of Technology, Harbin 150090, China; 19b933012@stu.hit.edu.cn

**Keywords:** trussed steel and concrete box beam, structural stressing state, numerical shape function, generalized strain energy density, failure load

## Abstract

This paper analyses the characteristics of the mechanical behavior of a trussed steel and concrete box beam under bending conditions based on the structural stressing state theory and the numerical shape function method. Firstly, the parametric generalized strain energy density was introduced to characterize the structural stressing state of trussed steel stud concrete box girders, and the strain energy density sum was plotted. Then the Mann-Kendall criterion was used to discriminate the leap point of the curve change and to redefine the structural failure load. By analyzing the strain and displacement, the existence of a sudden change in the structural response during the load-bearing process was again demonstrated. Afterwards, the numerical shape function method was used to extend the strain data, and further in-depth analyses of strain/stress fields and internal forces were carried out to show in detail the working characteristics of each under load. Through an in-depth analysis from different angles, the rationality of updating the failure load was verified. Finally, the effects of different structure parameters on the evolution of the structural stresses of the members were analyzed in a transversal comparison. The analysis results of the stress state of a steel-concrete truss structure reveal the working behavior characteristics of a steel-concrete truss structure from a new angle, which provides a reference for the design of a steel-concrete truss structure in the future.

## 1. Introduction

Through a significant number of engineering practices, it has been found that adding section steel to concrete structures can effectively improve the load-bearing capacity, stiffness, and ductility of components [1,2], etc., which is of great significance for the construction of modern engineering structures. Hence, the steel reinforced concrete structure as a type of structure which puts the steel into the traditional reinforced concrete can be used in the construction of large-span structures to improve the bearing performance [3,4,5].

As early as the beginning of the 20th century, experts and scholars began the research on steel reinforced concrete to promote the application of this technology in engineering. Chen [6] analyzed the effects of sectional steel disposition, the thickness of the concrete cover, and the concrete strength on the torsional performance of angle steel concrete beams. Xu [7,8] applied prestress technology to steel-reinforced concrete beams. Through experiments and numerical simulation, it was found that the mechanical properties of prestressed steel-reinforced concrete beams are better than ordinary steel reinforced concrete beams. Kozlov [9] used a scale model test to explore the shear performance and normal stress of a single-span steel-reinforced concrete beam. It was found that the test results were in good agreement with the calculated data. Aiming at the problem of structural corrosion, Meng [10] discussed the application of stainless-steel concrete structures in depth. Wu [11] carried out a finite element analysis of the new steel-concrete composite Virender beam by using ABAQUS. The results showed that the bending capacity and deformation performance of the new steel-concrete composite Virender beam were greatly improved compared with the ordinary reinforced concrete beam. Based on the test results, Yong [12] proposed two new stiffness calculation methods for partially prefabricated steel-reinforced concrete beams. Nguyen [13] discussed the influence of the restraint of steel bars and steel in concrete on the behavior of steel-reinforced concrete beams after yielding. Yang [14] conducted an in-depth study on the shear capacity of steel-reinforced concrete through experiments and revealed the shear failure mechanism of steel-reinforced concrete beams. Xue [15] proposed a theoretical model for predicting the shear strength of steel-reinforced concrete deep beams and short columns and verified the accuracy and safety of the model calculations based on experiments. Jeong [16] tried to analyze the changes in the neutral axis of steel-reinforced concrete beams, using a strain compatibility analysis method and proved its efficiency by comparing experiments and analysis values. Hong [17] proposed a new method that could more accurately predict the working behavior of steel-concrete mixed composite precast beams. In addition, domestic and foreign scholars have also carried out research on the performance of steel-reinforced concrete structures under special conditions [18].

From the above literature, it can be seen that traditional steel concrete structures are simply superimposed on steel and reinforced concrete structures, which improves some of their properties but increases the construction process. The truss steel-reinforced concrete box girder is a new type of steel-concrete composite structure and it is necessary to study its mechanical properties. The mechanical property test of large components is expensive, and the experimental data have not yet been fully applied, resulting in a large amount of the invisible information on the structural working behavior characteristics being ignored. Due to the limitations of the test point arrangement, the data measured are often limited and the limited test data are not sufficient to support an in-depth analysis of the elements and are not conducive to further research into steel-reinforced concrete. Moreover, currently, the ultimate load capacity of a steel-reinforced concrete structure is usually predicted using a semi-empirical and semi-theoretical approach, which often leads to increased costs and unreasonable structural designs based on safety considerations.

In order to gain a deeper understanding of the mechanical properties of the truss-type steel-reinforced concrete box girder, this paper attempts to further reveal the working characteristics of truss-type steel-reinforced concrete box beams subjected to bending loads by applying the structural stressing state theory. Then, the response data (strain, displacement, etc.) of beams are constructed and drawn, so that more of the stressing state changing characteristics of the beams can be analyzed from the curves in depth. The Mann-Kendall criterion is used to differentiate the characteristic loads, and the strain/stress fields and internal forces constructed based on the NSF method are used to further analyze the structural performance evolution characteristics. Based on the limited test data, this paper analyzes the force evolution process of the structure in depth, reveals the sudden change characteristics of the response, and provides a reference for the improvement of the structural design in the future.

## 2. Theory of Structural Stressing State Analysis

### 2.1. Method of Modeling a Structural Stressing State

The description of the structural stressing state on a structure is important to effectively reflect on its changing characteristics under load. In nature, everything, including a structure, changes according to the law of quantitative to qualitative change, and when the qualitative change occurs, things will deviate from their previous trajectory and enter a completely new stage of development. The response of a structure usually includes displacement, strain, load, and failure image. In contrast, displacement and strain are the most direct embodiments of the change in the stress state of the structure and reflect the stress evolution process of structure to a certain extent. However, the displacements and strains embedded with directions, namely vectors, affect the accuracy of the numerical model expression. Hence, the generalized strain energy density (GSED) [19] associated with stress and strain is proposed to describe the structural stressing state, and an analysis of the direction influences is also avoided due to the converting of a vector into a scalar. The formula for the GSED of the *i*-th position of the *j*-th load step can be expressed as:

(1) $$E_{ij}=\int_{0}^{\varepsilon_{ij}}\sigma_{ij}d\varepsilon$$

where $E_{ij}$ is the GSED value of the *i*-th element of the *j*-th load step and $\sigma_{ij}$ is the legal stress of the *i*-th position of the *j*-th load step andis the legal strain of the *i*-th position of the *j*-th load step. The stressed structural state of the whole structure can be expressed by accumulating the GSED values of each part with the following equations:

(2) $$E_{j}=\sum_{i=1}^{N}E_{ij}A_{i}$$

where *E_j_* is the GSED value of the section measured in the *j* load step and N is the total number of measurement points. In order to exclude the influence of the unit, the GSEDs are normalized into the massless *E_j_* and the norm is characterized by the stress state of the structure, as follows:

(3) $$E_{j,norm}=\frac{E_{j}}{E_{M}}$$

where $E_{j,norm}$ is the load *F_j_*’s normalized GSED and *E_M_* is the largest GSED and the entire loading process. By constructing this parameter, the *E*-*F* curve can be drawn to describe the stress variation characteristics of the structure.

### 2.2. The Application of the Mann-Kendall Criterion

The Mann-Kendall (M-K) criterion is a nonparametric method commonly used in trend analysis. This is a method used to reasonably infer the form of population distribution by using sample data. The Mann-Kendall test can be applied to determine whether there is a mutation in the sequence and if it exists, the time when the mutation occurs. In order to find the mutation point of the structural force state through the *E_j_*-*F* curve, the M-K method in statistics was introduced into the structural force state analysis. It was assumed that the {*E(i)*} sequence (load step *i* is 1, 2, …, n) was statistically independent, based on the curve, which defines the cumulative number *m_i_* as:

(4) $$m_{i}=\left\{ \begin{array}{l} +1,E_{i}>E_{j}(1\leq j\leq i)\\ 0,otherwise \end{array}\right.$$

where “1” means that if the inequality on the right side of the *j* comparison is satisfied, 1 is added to the existing value. The *k* load step is then defined with a new random variable *d_k_*:

(5) $$d_{k}=\sum_{i=1}^{k}m_{i},2\leq k\leq n$$

The mean value *E(d_k_)* and variance *Var(d_k_)* of dk are calculated by:

(6) $$E(d_{k})=\frac{k(k-1)}{4},2\leq k\leq n$$

(7) $$Var(d_{k})=\frac{k(k-1)(2k+5)}{72},2\leq k\leq n$$

Then, a new statistic *GF_K_* is defined by:

(8) $$GF_{k}=\frac{d_{k}-E(d_{k})}{\sqrt{Var(d_{k})}},\;2\leq k\leq n$$

From this, the *GF_k_*-*F_j_* curve can be obtained, and then the *GB_k_*-*F* curve can be formed by applying the same process to it in reverse. The two curves can intersect at the mutation point of the *E_j_*-*F_j_* curve, thus generating the identification structure stress state criteria for the transition points.

## 3. Introduction of the Experiments

### 3.1. Specimen Design

**Figure 1:**
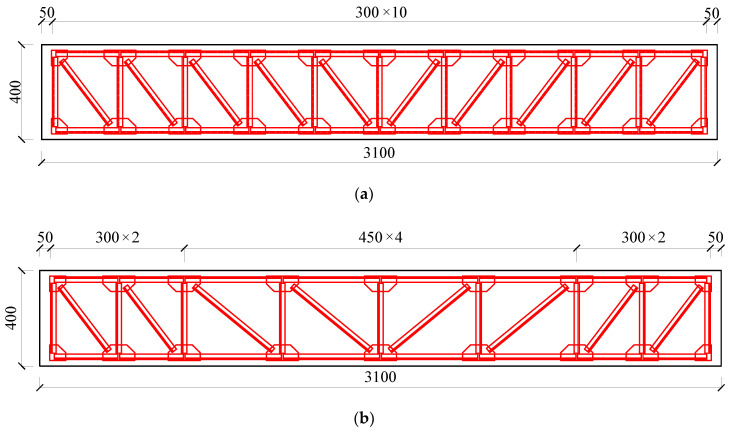

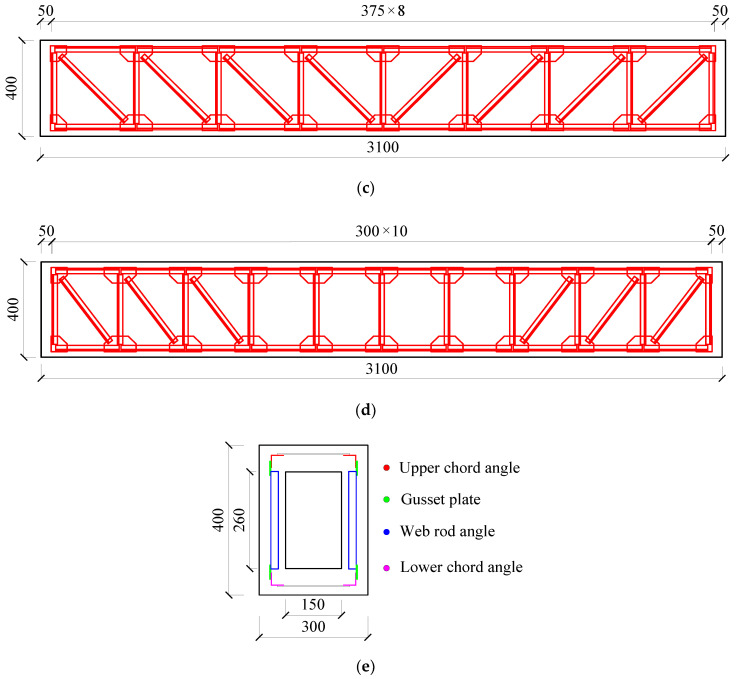
Design schematic: (**a**) SRC-A1~A3 longitudinal section; (**b**) SRC-A4 longitudinal section; (**c**) SRC-A5 longitudinal section; (**d**) SRC-A6 longitudinal section; (**e**) Midspan section of specimen. (Unit: mm).

Liu Qiang [20] designed the fabrication of six test beams for testing and verifying the performance of truss-type steel-reinforced concrete box beams, as shown in Figure 1. In order to ensure that the flexural failure of the box girder conforms to the failure mode of the appropriate reinforcement beam, the truss joint adopts a thickened gusset plate and three side circumferential seam welding to improve the bearing capacity of the joint. The concrete grade is C30, the measured compressive strength of concrete cube is 30.9 MPa. The modulus of elasticity is 3.00104 GPa, the precast truss steel is Q235, and the measured values of mechanical properties are shown in Table 1, the thickness of angle protection layer is 30 mm, and other specific parameters of the six beams are shown in Table 2.

### 3.2. Measurement Point Arrangement and Loading Scheme

**Figure 2:**
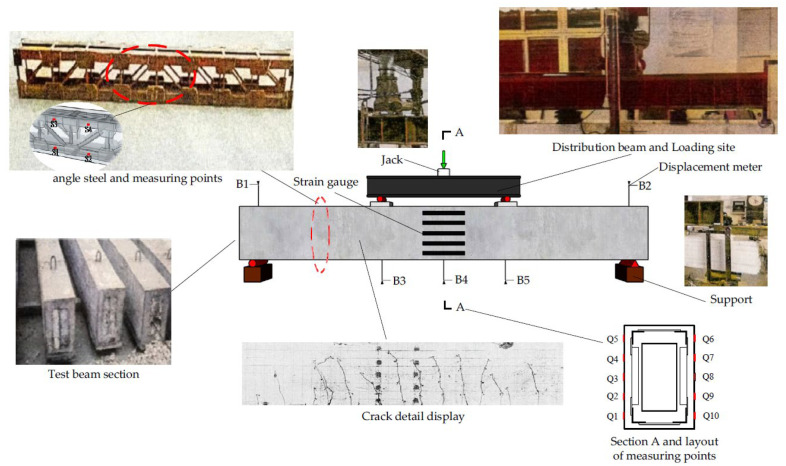
Schematic diagram of the experimental setup.

The loading test method and measurement points were designed as shown below in Figure 2. The test specimens were simply supported specimens with a span of 3100 mm. The three-point loading was adopted, and the strain of angle steel was measured by a TS3890 static resistance strain gauge (S1-S4). The model of the resistance strain gauge was BFH120-3AA-D100, the resistance value was 120 Ω, the sensitivity coefficient K = 2.0 ± 1%, and B1-B5 were dial indicators (accuracy: 0.01 mm) to measure displacement. The strain gauge was used to measure the displacement of the loading point, span, and support, respectively. Q1-Q10 were the dial indicators (accuracy: 0.001 mm) to measure the concrete surface strain. They were symmetrically distributed, with an upper and lower spacing of 75 mm as shown in Figure 2.

The six beam specimens in this test were simply supported specimens, that is, one end of the support was a fixed hinge support, one end was a movable hinge support, and 100 mm was reserved at both ends of the support to prevent the support from sliding. The test loading method adopted three-point loading, which was realized through the distribution beam and Jack. The load was controlled by the pressure sensor. Steel plates were padded at the loading point and supported to increase the local compression area. The pre-loading test was firstly carried out to check the test apparatus and to reduce the experimental errors brought about by the test apparatus, and in the formal loading stage, the beams were loaded at 10 kN per stage until failure.

## 4. Analysis of the Structural Stressing State of the Beam-A3

### 4.1. The E_j_-F_j_ Curve of the Test Beam and Failure Load Analysis

**Figure 3:**
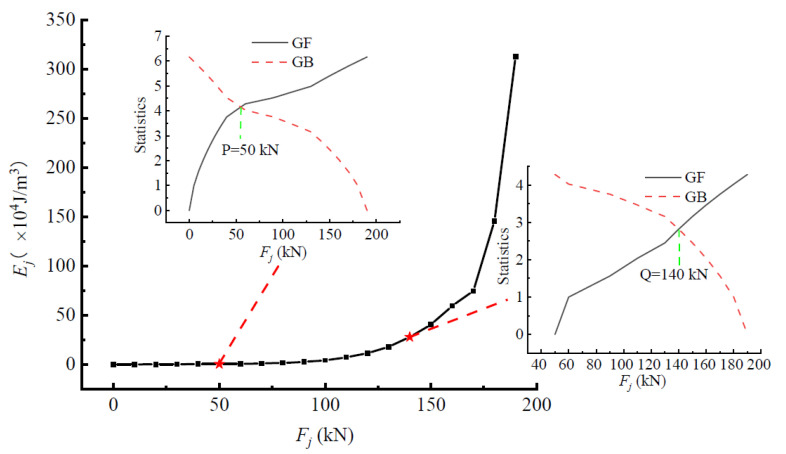
The *E_j_*-*F*_j_ and M-K statistic curves of the SRC-A3 beam.

In this section, taking the A3 beam as an example, the variation characteristics of stress state of the structure during the whole loading process are analyzed by using the above theoretical methods. The *E_j_*-*F_j_* curve of beam-A3 and the characteristic points *P* and *Q* corresponding to the intersection of *GF*-*F_j_* and *GB*-*F_j_* curves obtained by the M-K method were drawn as shown in Figure 3. It can be seen that the development of the *E_j_*-*F_j_* curve was roughly divided into three stages by the two characteristic points *P* and *Q*. Before the load reached the *P* point, the *E*-*F* curve changed very gently, which indicates that the concrete was not cracked at that time, and the beam was basically in a relatively stable linear elastic stage. The tensile performance of the concrete was very poor, which was far lower than its compressive performance. Therefore, with the increase in load, the concrete in the tensile area will soon reach its tensile strength limit, resulting in the first cracking. Between *P* and *Q*, the curve raised slightly, and more cracks appeared in the tensile zone, and the stress of the tensile angle steel increased. The tensile stress would be transferred to the uncracked concrete through the cohesive force leading to some new cracks and extensions of existing cracks. Although the corresponding *E_j_*-*F_j_* curve never increased in line and the beam entered the elasto-plastic stress stage, it still maintained a stable stress state macroscopically. Compared with the linear elastic stage, the beam began to develop in a part of the plasticity and experienced local damage, and it could not completely restore its original state despite removing the load. After the *Q* point, the curve became very steep and the beam entered the unstable damage phase, leading to large deformations at very small load increments, which are not conducive to continuous loading until the final failure. This abrupt change reveals that the structural stress state of the beam jumped from the previous elasto-plastic state to a new unstable developing stress state, where the beam will not recover after force deformation. In other words, after the *Q* point, the structural stress state of the beam began to change qualitatively.

The first characteristic point *P* here reveals the transition of the structure from the elastic stress state to plastic development, which is the transition from concrete never cracking to cracking. After point *P*, the structure entered the elastic-plastic stress stage. The whole structure maintained a stable stress state macroscopically before the characteristic load *Q*, and the stress state of the beam always maintained a quantitative change rather than a qualitative change. The second characteristic point *Q* reveals that the structure changed from the previous elastic-plastic state to the new stress state of unstable development, and the members will no longer recover after stress and deformation. This is the inevitable working behavior feature of the structure in the stress process; that is, the stress state of the structure changed qualitatively at point *Q*, and the load at point *Q* was the starting point of the structural failure process and the critical point in the change process of the stress state of the members. After the *Q* point, the structure began to enter the failure development stage. The determined critical load was the result of the internal mutation of the characteristics of the stress state of the structure, not the assumption. The whole process of the stress state change represents the inherent leap characteristics of the structural stress state, which reveals the natural law of stress state development after structural stress. In other words, the sudden change in the load of the stress state of the truss steel-reinforced concrete beam determined by this method can be used as a reference for determining the design load of the truss steel-reinforced concrete structure.

According to the analysis of the development characteristics of the *E_j_*-*F_j_* curve, it was found that the characteristic points *P* and *Q* played important roles in the loading process. These can be summarized as follows: (1) Characteristic point *P* represented the transition point of the elastic stressing state to the plastic development for the beam. (2) Characteristic point *Q* was the starting point of the structural failure and was the critical point in the process of qualitatively changing the structural stressing state of the beam, which essentially reflected the internal law of the structure under load.

### 4.2. Strain-Based Characterization of the Stressing State for Beam-A3

**Figure 4:**
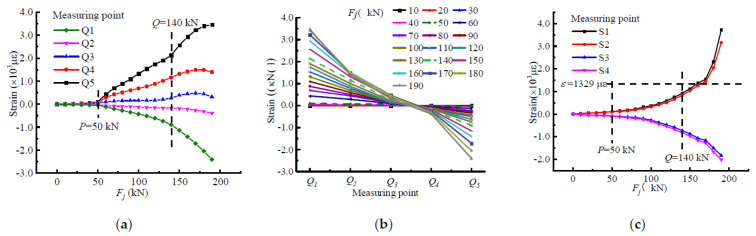
Strain-based stressing state analysis of beam-A3: (**a**) *F_i_*-ε*_i_* curve; (**b**) ε*_i_*-*Q_i_* curve; (**c**) angle steel strain-load curve.

Figure 4 shows the change in the measured strain value of beam A3 with load, and a strain pattern diagram was added to show the strain change more vividly before and after loading. The characteristic loads are indicated by dashed lines, and the results show that the curves had the same trend as the *E_j_*-*F_j_* curve, which was divided into three different stages of development by points *P* and *Q*. As can be seen from the curve of strain versus load, the strain values at each measurement point were usually very small before the characteristic point *P*. The curves almost overlapped, were very close to each other, and remained essentially linear. It can be seen that the beam was in a state of elastic stress and the tensile strain of the concrete at the tensile edge had not yet reached the ultimate tensile strain. After that, the strain curve of the beam grew significantly and separated, and the beam entered the plastic development stage. The tensile zone was subjected to angular tensile stresses and the concrete was withdrawn from the works. From characteristic point *P* to *Q*, the strain increased more rapidly compared to the previous stage. The angle steel had good mechanical properties, and the outsourced concrete could effectively prevent the local buckling of the angle steel, which could effectively control the structural deformations. Therefore, the stress-strain curve changed smoothly. When it was over the characteristic load *Q*, the curves changed in unstable trends, which shows a certain abrupt change characteristic. The beam began to be in a state of instability and stress and entered the failure development stage. Eventually, the concrete in the compression zone was crushed and the beam was destroyed.

### 4.3. Displacement-Based Characterization of the Stressing State for Beam-A3

**Figure 5:**
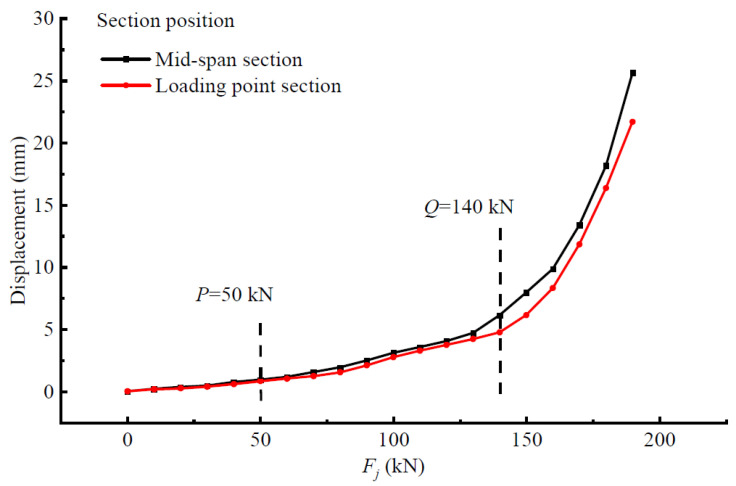
Displacement-*F_j_* curve.

The changing characteristics can also be reflected in the displacement to a certain extent; hence, the displacement-load curve at the mid-span and loading point of beam-A3 is drawn in Figure 5. In order to show the change law more vividly, the mid-span displacement increment diagram was supplemented. The displacement curve can reflect the change trend of displacement before and after a two-level load. With the increase in the load, the displacement of the mid-span section was greater than that of the loading point, which was consistent with the stress and deformation characteristics of the structure. In addition, the displacement and its incremental curve had similar variation characteristics, which can verify the correctness and validity of the three stress state stages divided by the two characteristic loads.

## 5. Stress State Analysis Based on Strain Interpolation

In structural analysis, the behavior state characteristics of the structure are often reflected by the measured data. The description of the response of the measured data to the structure is scientific and reasonable, and it is also one of the most accurate methods. However, due to the limitation of measuring instruments, measuring conditions, and methods, the measured data are limited and the finite data are often not enough to prove the response mechanism and state characteristics of the structure. Therefore, a method with precise physical significance is needed to expand the test data to obtain more information about the stress state of the structure. The numerical shape function interpolation (NSF) [21] method emerges.

### 5.1. Numerical Shape Function Method

The current interpolation method does not consider the specific physical model and is mainly used for an internal supplement when the data are missing. The NSF method is based on the concept of shape function in the finite element method. It uses experimental data as weights and node data as the basis. The relevant data field of the entire component is obtained through the method of interpolation, and then the basic configuration and configuration of the force state of the entire structure are determined. It is a kind of numerical shape function based on a finite element numerical simulation and the concept of shape function to construct a numerical shape function in line with the physical characteristics of the model. It can not only overcome the shortcomings of traditional interpolation methods, but can also obtain data close to the real test data field to ensure the accuracy of the in-depth test and analysis.

**Figure 6:**
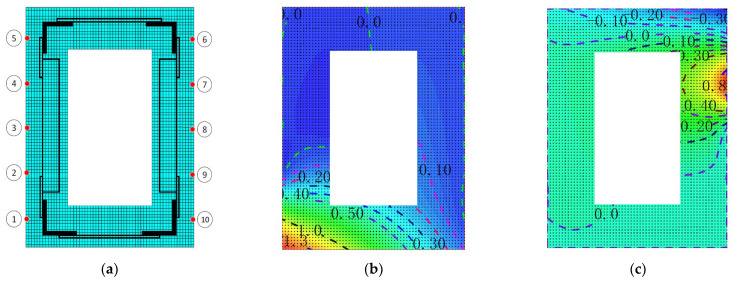
Finite element model and contour maps of numerical shape functions: (**a**) the square plate model; (**b**) shape function N1; (**c**) shape function N7.

In order to introduce this method, the large-scale general ANSYS [22] software is used for modeling and meshing. The element type is shell 181 and the size is 5 mm, as shown in Figure 6a. Then the *z*-axis unit strain is applied at a corresponding measuring point to the section, and z-directional constraints are imposed on other nodes, limiting its rigid body displacement, and then a static analysis is performed to obtain the z-directional strain field at the corresponding measurement point, as shown in Figure 6b,c. According to Castigliano’s theorem, in this case, the constructed strain field is independent of the load path, and the results of the simulation can be linearly superimposed. Similarly, Formula (9) can be used to obtain the strain field of the entire model:

(9) $$D=\sum_{i=1}^{m}u_{i}N_{i},N_{i}=[N_{i}(x_{1}),N_{i}(x_{2}),\ldots N_{i}(x_{j})\ldots N_{i}(x_{n})]$$

where *D* is the deflection field of the section, *N_i_* is the numerical shape function of the *i* measuring point, *N_i_(x_i_)* is the function value of the element node *x_j_*, *n* is the total number of element nodes, and *m* is the total number of element nodes.

**Figure 7:**
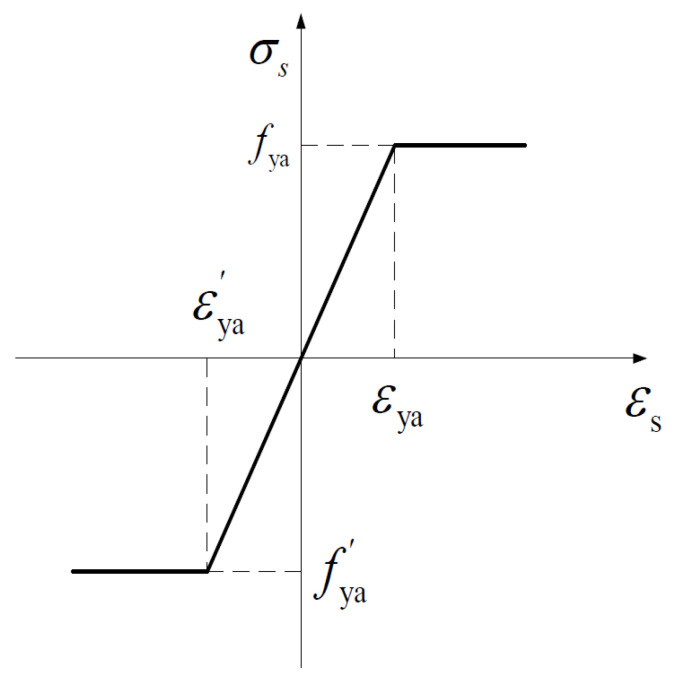
Stress-strain relationship of angle steel.

When constructing the constitutive relationship, the compression constitutive curve of concrete is [23]:

(10) $$\{\begin{array}{l} \sigma =f_{c}\cdot \left[\alpha_{a}\frac{\varepsilon }{\varepsilon_{c}}+\left(3-2\alpha_{a}\right)\cdot {\left(\frac{\varepsilon }{\varepsilon_{c}}\right)}^{2}+\left(\alpha_{a}-2\right)\cdot {\left(\frac{\varepsilon }{\varepsilon_{c}}\right)}^{3}\right],\varepsilon \leq \varepsilon_{c}\\ \sigma =f_{c}\cdot \frac{\frac{\varepsilon }{\varepsilon_{c}}}{\alpha_{d}{\left(\frac{\varepsilon }{\varepsilon_{c}}-1\right)}^{2}+\frac{\varepsilon }{\varepsilon_{c}}},\varepsilon >\varepsilon_{c} \end{array}$$

where $f_{c}$ is the axial compressive strength of concrete (N/mm^2^); $\varepsilon_{c}$ is the peak compressive strain of concrete corresponding to $f_{c}$; $\alpha_{a}$ is the parameter value of the stress-strain rising section under uniaxial compression, and $\alpha_{d}$ is the parameter value of the stress-strain drop section under uniaxial compression. The compression constitutive curve of concrete is:

(11) $$\{\begin{array}{l} \sigma =f_{t}\cdot \left[1.2\frac{\varepsilon }{\varepsilon_{t}}-0.2{\left(\frac{\varepsilon }{\varepsilon_{t}}\right)}^{6}\right],\varepsilon \leq \varepsilon_{t}\\ \sigma =f_{t}\cdot \frac{\frac{\varepsilon }{\varepsilon_{t}}}{\alpha_{t}{\left(\frac{\varepsilon }{\varepsilon_{t}}-1\right)}^{1.7}+\frac{\varepsilon }{\varepsilon_{t}}},\varepsilon \geq \varepsilon_{t} \end{array}$$

where $f_{t}$ is the axial tensile strength of concrete (N/mm^2^); $\varepsilon_{t}$ is the peak compressive strain of concrete corresponding to $f_{t}$, and $\alpha_{t}$ is the parameter value of uniaxial tension descending section. Moreover, the constitutive curve of angle steel is shown in Figure 7, and the relationship expression is shown in formula 12.

(12) $$\sigma_{s}=\{\begin{array}{cc} f_{ya} & \varepsilon_{ya}\leq \varepsilon_{s}\\ E_{s}\varepsilon_{s} & ,\;\varepsilon_{ya}^{\prime }<\varepsilon_{s}<\varepsilon_{ya}\\ f_{ya}^{\prime } & \varepsilon_{s}\leq \varepsilon_{ya}^{\prime } \end{array}$$

where $f_{ya}$ is the tensile yield strength of angle steel (N/mm^2^); $f_{ya}^{\prime }$ is the yield strength of compression angle steel (N/mm^2^); $E_{s}$ is the elastic modulus of angle steel (N/mm^2^): $\varepsilon_{ya}$ is the tensile yield strain of angle steel, and $\varepsilon_{ya}^{\prime }$ is the compressive yield strain of angle steel.

### 5.2. Extended Data Accuracy Analysis

**Figure 8:**
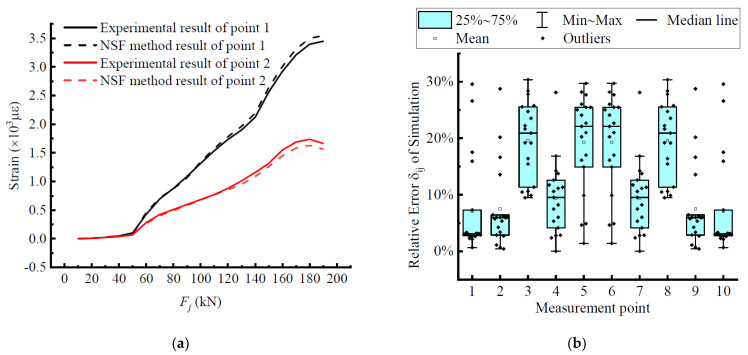
Error analysis of the NSF method: (**a**) the comparison between the experimental and interpolated strains; (**b**) the relative error of the measurement point.

Take measurement points 1 and 2 as examples to clearly observe the fitting degree between the interpolation data and measurement data, as shown in Figure 8. It can be clearly seen in Figure 8a that the two curves achieved a high degree of fit throughout the loading process and even overlapped in most stages, which indicates that the data derived from applying this difference method have a fairly high accuracy, a high degree of fit to the experimental results, and relatively small errors, which can meet the application requirements. Figure 8b shows the statistical information of its errors represented by a box-line plot, where the fit can be more obviously seen. The height of the quadrature spacing boxes of measurement point 1 and measurement point 2 was relatively small, so the data show a certain concentration phenomenon and the average error was small. It echoes with the previous curve fitting results, which further verifies the rationality and scientific effectiveness of the NSF interpolation method. By comparison, all measurement points were within the error tolerance range, indicating that the extension of the test data by this interpolation method is scientific and reasonable, and is an extension of the structural analysis method, which can be used as an important tool for analyzing the stress state of beams.

### 5.3. Strain/Stress Field Analysis

The experimental data can reflect the performance characteristics of precast truss-type steel-reinforced concrete around the sudden load to a certain extent, but the limited data can only reflect the strain/stress distribution and development of each measurement point. The NSF method was used to expand the experimental data of the section of the beam, and then stress was calculated based on the constitutive relation model of materials, including concrete and steel. Therefore, the strain/stress fields of the section were constructed and used to reveal the changing characteristics of the structural stressing state of the beam. By integrating the data obtained, the evolution process of the structural stress state under the vertical load in the mid-span section of the component is displayed, and the jump process of the structural stress state before and after the characteristic point was verified, which can intuitively show the structural stressing state in the loading process.

**Figure 9:**
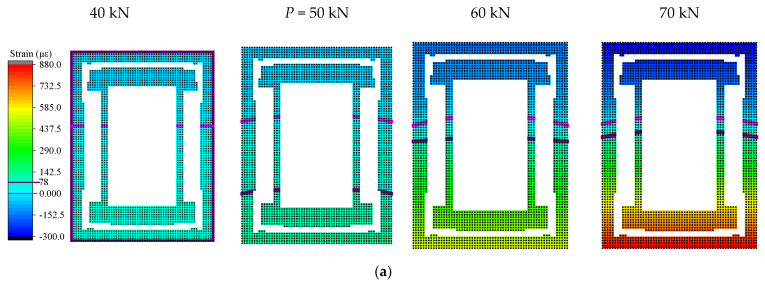

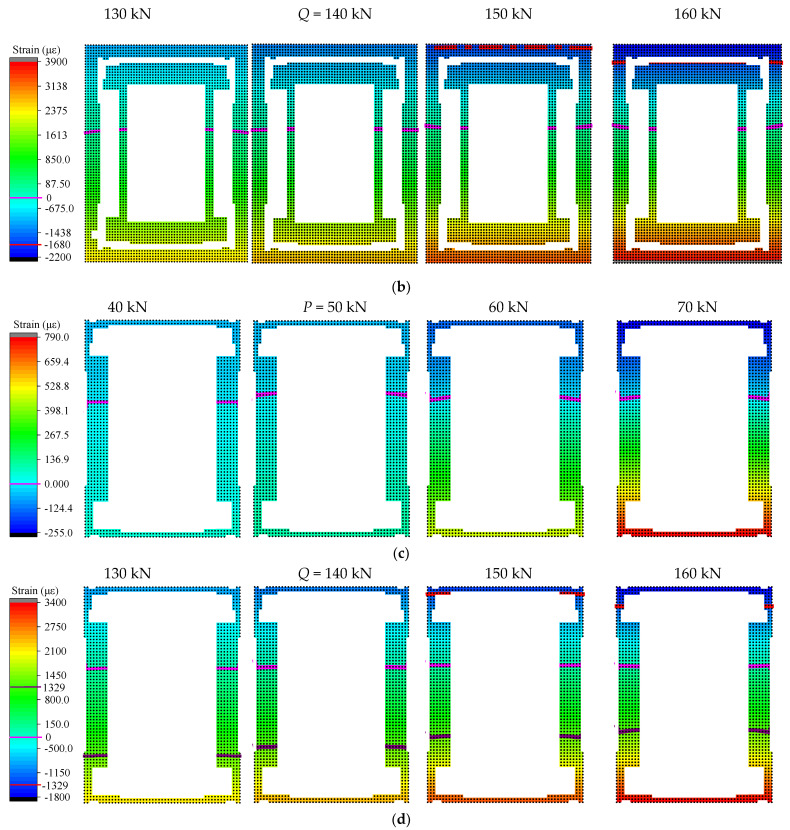
Strain fields of concrete and angle steel around the characteristic loads *P* and *Q*: (**a**) strain fields of the concrete around load *P*; (**b**) strain fields of the concrete around load *Q*; (**c**) strain fields of angle steel around load *P*; (**d**) strain fields of angle steel around load *Q*.

Figure 9 depicts the concrete strain field diagram obtained by the interpolation method near the *P*-value and *Q*-value load of the mid-span section, and the same section uses the same colorimetric and scale, marking the main grade scale value and 0 value position on the scale. The boundary line where the strain is 0 is marked with a magenta line, the peak tensile strain is marked with a purple line, and the peak compressive strain is marked with a red line. The specimen was directly subjected to the downward load, and the cross-section presents a state of upper compression and lower tension. The red area indicates the maximum tension, and the blue area indicates the maximum compression. It can be seen that the area of the tension zone of the entire cross-section was larger than the compression zone. Before the load *P*, the color of the strain field was lighter, and the concrete had not yet reached the ultimate tensile strain, meaning that the concrete was still in the elastic working stage at this time, and no cracks had occurred. When the load was at the *P*-value, due to the appearance of cracks, the tensile strain peak line appeared on the cloud diagram. Compared with the previous, the strain field changed significantly, and the peak line gradually moved upward with the increasing load, leading to the upward development of the crack. It can be concluded that the stress state of the structure changed after 50 kN, but the development of the strain field remained relatively stable. In addition, in Figure 9d the appearance of the peak line on the figure of the angle steel indicates that the tension zone had reached the tensile strain at the yield, and as the load increased, the position of the peak line gradually moved upward. As the neutral axis moved upward, more and more concrete lost its ability to resist tension, as the tensile stress on the angle steel continued to increase. After the limit value and *Q* value, the color of the concrete gradually darkened, and a compressive strain peak line appeared, indicating that the force state of the member was no longer stable at this time. The maximum tensile/compressive strain value of the cross-section increased with the increase in the load, the tensile area was continuously reduced, and its changed forms all had a sudden change after the *Q* value. The compressive strain peak line of the angle steel was also generated, and the super large strain value appeared near the lower part of the section. The compressive strain peak line of the angle steel was also generated, and the super large tensile strain value appeared near the lower part of the section, which shows that the structure was in an unstable state and had potential risks. The structural stress state of the beam jumped, and the continuous loading entered the failure stage. Finally, the concrete in the compression zone was crushed, resulting in the complete failure of the beam.

**Figure 10:**
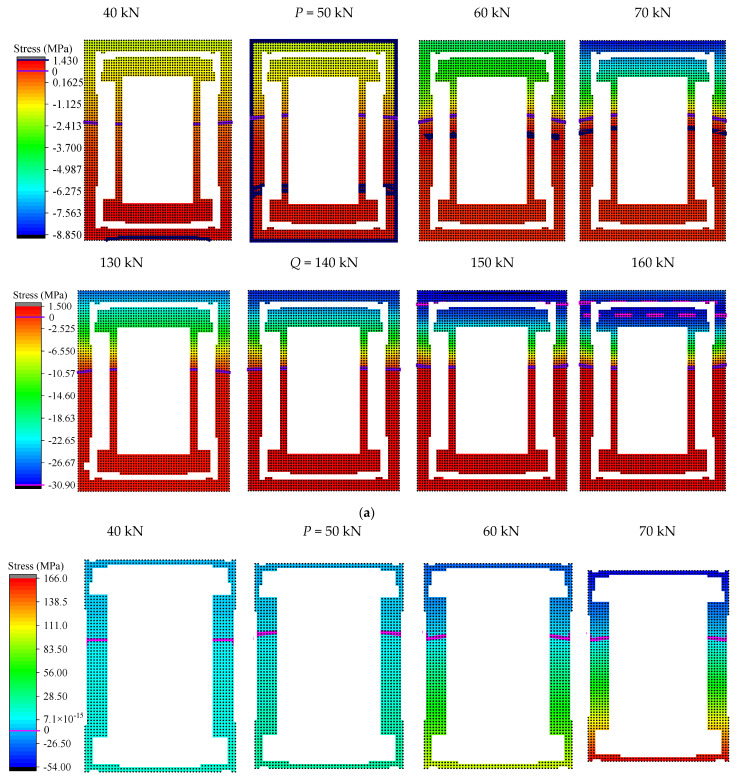

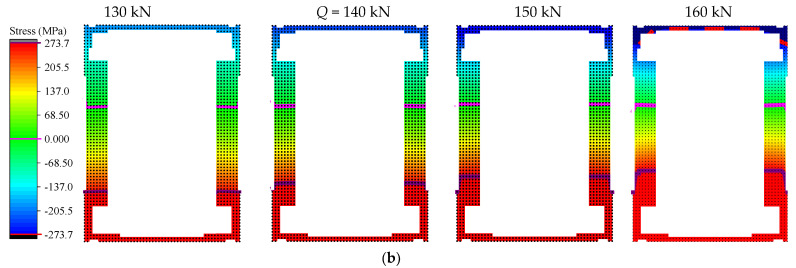
Stress field around the characteristic loads *P* and *Q*: (**a**) strain field of concrete around loads *P* and *Q*; (**b**) strain field of angle steel around loads *P* and *Q*.

In order to further observe the changing characteristics of the structural stressing state around the characteristic loads, the stress fields of concrete and angle steel were plotted in Figure 10, respectively. The maximum compressive stress of concrete was 30.7 MPa, which was close to the compressive strength of the cube. The corresponding peak line was generated after load *Q* and developed downward from the upper edge of the section. After load *P*, the minimum tensile stress in the concrete reached a maximum value (1.43 MPa), resulting in cracks. From then on, the concrete in the tensile zone stopped working and the stresses were redistributed. Some characteristics of the changes in the stress field can be observed before and after load *P*. The tensile stress in the angle continued to increase with the increase in load. In the concrete stress field, it can be seen that as the load continued to increase, the area surrounded by the maximum tensile strain peak line of the concrete and the strain line with a strain of 0 gradually decreased. After load *Q*, the stress field was characterized by abrupt changes, such as the compressive zone of concrete and the tensile zone of angle steel. Through the above, it was found that loads *P* and *Q* could indeed define the change characteristics of the structural stressing state for the beam more accurately.

### 5.4. Internal Forces Analysis

**Figure 11:**
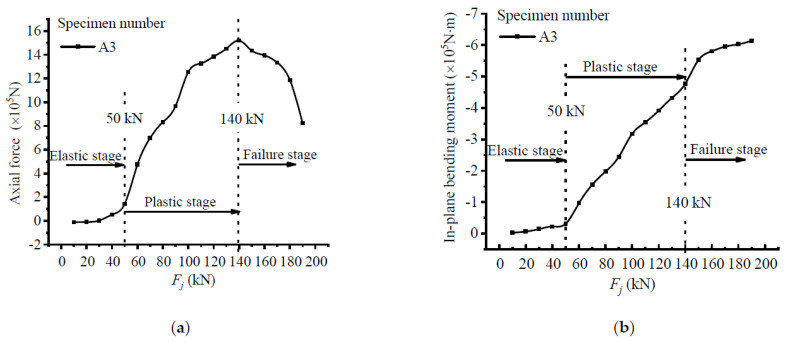
Section force characteristic curve: (**a**) axial force curve; (**b**) in-plane bending moment curve.

Under the action of a load, the test beam was mainly subjected to axial pressure and an in-plane bending moment, and they were separated from each other in order for the changing trend of the structural stressing state under different internal forces to be studied. The axial force and in-plane bending moment were direct manifestations of the structural stressing state, and they are plotted in Figure 11. It can be seen that the axial force first increased then decreased, while the in-plane bending moment occurred all the time. The maximum axial force and bending moment in the whole loading process were at load *Q* about 1500 kN and at the ultimate load about −740 kN, respectively. The sudden deviation of the changing trend for the two curves can also be clearly identified before and after loads *P* and *Q*; hence, three structural stressing state stages were divided, which were the elastic stage, plastic stage, and failure stage, respectively. The changing characteristics are shown in the *E_j_*-*F_j_* curve and once again verify the previous discovery.

## 6. Effects of Different Test Parameters on the Stress State Characteristics of a Steel Stud Concrete Box Girder

### 6.1. Analysis of Different Truss-Type Steel Stud Concrete Stress State Patterns Based on GSED

**Figure 12:**
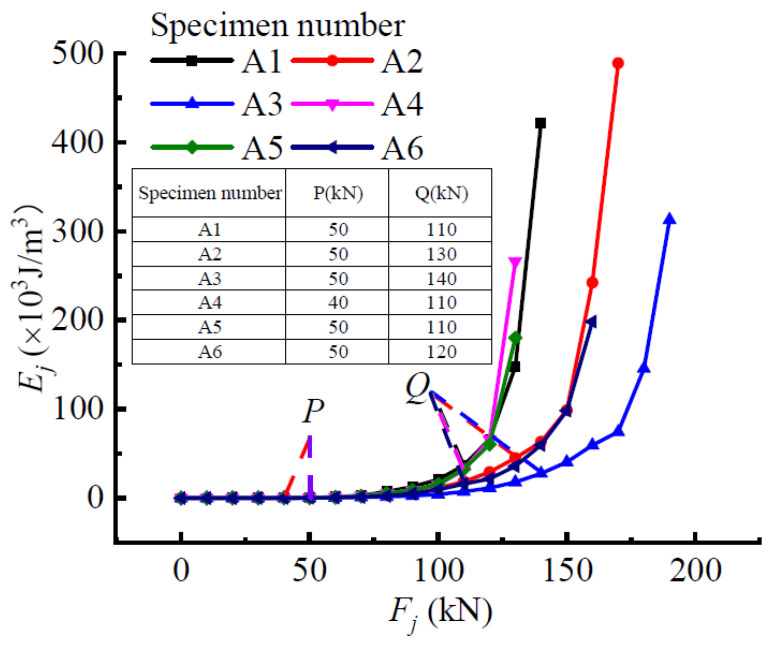
*E_j_*-*F_j_* curve.

With reference to the analysis method of beam-A3, the evolution law of the structural stressing state of other test beams under the same loading conditions were compared and analyzed in turn. Figure 12 shows the *E_j_*-*F_j_* curves of all the beams, and it is still obvious that the curve change in the entire loading process can be divided into three stages by their respective characteristic loads. The magnitude of the lower chord angle was an important factor affecting the strain energy response of the members, but by comparing loads *P* for A1, A2, and A3, it had almost no effect on the elastic-plastic boundary point of the angular concrete beam, indicating that the determination of the mutation point may be related to the material properties of the concrete only. As for the second load *Q*, its value increased as the size of the lower chord angle steel increased. In other words, the change in the size of the bottom chord angle steel can affect the failure load of the component; thus, the larger the size, the greater the bearing capacity.

The vertical web spacing determines the number of vertical webs and diagonal webs in pure bending, and the number of webs has an effect on the concrete restraint. Comparing the three beams A1, A4, and A5 with different web bar spacing, it can be found that their *Q* values were all 110 kN, but the values of load *P* reduced with the decrease in the vertical web rod space. It can be indicated that the distance between the vertical web members of the pure bending section had little effect on the flexural bearing capacity of the truss-type steel-reinforced concrete box girder, but it affected the elastic phase of concrete to some extent, such as negatively affecting the crack resistance.

Compared with A2 and A6, the only difference between them is whether there was an oblique web angle steel in the pure bending section, which generally bears the shearing effect in the specimen. In the *E_j_*-*F_j_* curve, the *P* values were both 50 kN, and the load *Q* of specimen A6 was 120 kN, which was slightly smaller than that of specimen A2 of 130 kN. Although there were some differences between the two beams, the influence of oblique web members on the stressing state of the truss-type steel-reinforced concrete box girder under the same load was not obvious.

### 6.2. Analysis of Strains and Displacements of Different Steel Reinforced Concrete

**Figure 13:**
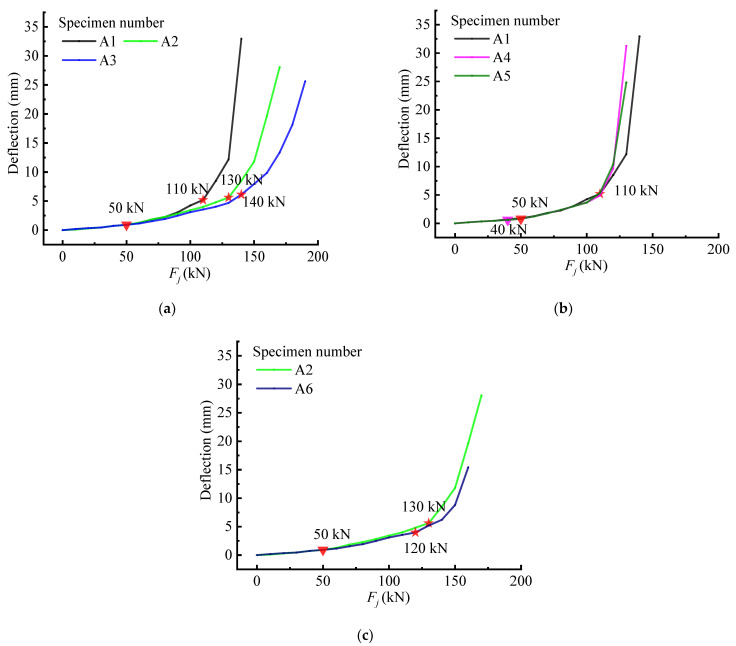
Mid-span displacement of the beams: (**a**) A1\A2\A3 beam; (**b**) A1\A4\A5 beam; (**c**) A2\A6 beam. (The inverted triangle is the *Q* value and the pentagram is the *P* value, different colors represent different specimens).

In order to further study the influence of changes in steel frame design parameters on the performance of beams under load, the following figure summarizes the changes of relevant displacement and strain of beams with three groups of different steel frame parameters with load. The displacement can reflect the change characteristics of the stress state of the beam in another way. It can be seen in Figure 13 that the changing characteristics of displacement were similar to those of GSED, and the characteristic loads and stressing state stages could also be separate from the curves, which can also verify the accuracy and efficiency of the M-K method. However, there were still some differences between them around ultimate loads, which could reflect the changing characteristics of different types of beams during the failure stage. It can be seen from A1-A5 that the maximum displacement would increase with the increase in web bar spacing and the decrease in the distance between vertical web members, but they had a similar displacement at characteristic loads. Through a comparison with A2 and A6, it can be also found that the oblique web angle steel in the pure bending section could effectively improve the ductility and bearing ability.

Due to the lack of a qualitative analysis of structural failure, the current structural design generally adopts measures such as the excessive use of materials to improve the safety factor, and the failure process of the structure cannot be accurately controlled. The theory of the structural stress state is of great significance to judge the damage to structures and can provide a reference for structural design. In structural health monitoring, it is necessary to control the whole process of the structural response. The scientific evaluation of the structural bearing capacity is the focus of structural health monitoring. The application of this method can also be used as the basis to judge whether the structure can be used normally. In structural analysis, the critical point of the structural response to qualitative change can be scientifically evaluated by using this method, which can be used as an important index to evaluate the mechanical properties of structures.

Based on the research idea of this paper, through the analysis of the whole process of the structure and optimizing the seismic analysis model, it is helpful to judge the working state of the structure under different ground motion levels. Moreover, based on the theoretical method of this paper, expanding the test data is helpful in order to analyze the overall performance of the structure in depth and to scientifically predict the failure position, elastic-plastic deformation degree, and damage degree of the component.

## 7. Conclusions

In this study, six steel truss-reinforced concrete box beams with different parameters were introduced. Based on the structural stressing state theory, the stressing state changing characteristics of the specimens during the entire loading process were analyzed, and a new Generalized Strain Energy Density (GSED) parameter was introduced to describe the response of the structure. The application of the M-K criterion revealed the jump characteristics of the structural stress state and redefined the failure load. The test data were extended by the NSF method to ensure the accuracy of the in-depth analysis of the structural test. Based on the interpolation data of the NSF method, the stress/strain field of the section was analyzed, which further reflected the sudden change in the internal stress state of the member before and after the characteristic load and verified the rationality of the M-K criterion. In addition, the stress state sub-mode of internal force showed the development trend of the axial compression and the in-plane bending moment. Finally, through a horizontal comparison, the influence of different structural parameters on the characteristic load of the specimen was shown. The results obtained improved the lack of test data and improved the accuracy of analysis. The results provide a new method for test data processing and analysis. The characteristic load judged according to the M-K criterion reflected the inherent characteristics determined in the process of the structural work. The failure load was accurately determined, which can provide a technical reference for the design of truss-type steel-reinforced concrete in the future.

In future work, I will continue to compare the structural performance of a steel-concrete composite truss box girder and conventional structures through relevant experiments and numerical simulation, and analyze the performance advantages of reinforced concrete composite truss structures compared with ordinary structures in depth. In order to meet the seismic requirements, we will further explore the ductility performance, and provide an in-depth analysis of the seismic performance of a steel-concrete composite truss structure under earthquake (such as structural damage resistance, deformation capacity, energy dissipation capacity, etc.) by means of a test and a numerical simulation. This test adopts the form of simply supported members, and the connection mode of angle steel is welding. If conditions permit, the mechanical properties under other connection modes (such as the bolt connection) will be explored, and other forms of boundary conditions will be analyzed.

## Figures and Tables

**Table 1:** The measured values of the mechanical properties of angle steel.

Angle Steel Type	Specification	Yield Strength (MPa)	Ultimate Strength (MPa)	Elastic Modulus (GPa)
Equilateral angle steel	L30 × 3	273.7	379.5	206
	L40 × 4	306.4	438.7	192
	L45 × 5	290.8	407.6	198
	L50 × 5	279.2	393.8	200

**Table 2:** Specimen design parameters.

Specimen Number	Angle Steel				
	Upper Chord Angle	Lower Chord Angle	Vertical Web Rod Angle	Oblique Web Angle Steel	Vertical Web Bar Spacing (mm)
SRC-A1	L30 × 3	L40 × 4	L30 × 3	L30 × 3	300
SRC-A2	L30 × 3	L45 × 5	L30 × 3	L30 × 3	300
SRC-A3	L30 × 3	L50 × 5	L30 × 3	L30 × 3	300
SRC-A4	L30 × 3	L40 × 4	L30 × 3	L30 × 3	450
SRC-A5	L30 × 3	L40 × 4	L30 × 3	L30 × 3	375
SRC-A6	L30 × 3	L45 × 5	L30 × 3	No(pure bending section)	300

## Data Availability

Data are available on request to the authors.
